# Synergistic immunomodulatory effects of naringenin and photothermal therapy in triple-negative breast cancer

**DOI:** 10.1016/j.isci.2025.114124

**Published:** 2025-11-19

**Authors:** Chunyu Lei, Yu Yao, Qian Xie, Na Li, Chenghao Zhu, Haomiao Lan, Sisi Yang, Gang Wang, Xianfeng Peng, Duwen Zheng, Haili Wang, Ke Zhu, Ziyang Zhu, Fuqiang Shao

**Affiliations:** 1 Department of Nuclear Medicine, Zigong First People’s Hospital, Zigong Academy of Medical Sciences, Zigong 643000, China; 2 Department of Medical Ultrasound, Shanghai Tenth People’s Hospital, Ultrasound Research and Education Institute, School of Medicine, Tongji University, Shanghai 200072, China; 3 Department of Diagnostic Ultrasound, Wuhan Children’s Hospital (Wuhan Maternal and Child Healthcare Hospital), Tongji Medical College, Huazhong University of Science and Technology, Wuhan 430015, China; 4 Department of Ultrasonogrphy, Zigong Fouth People’s Hospital, Zigong 643000, China; 5 Medical Department, Wuhan City College, Wuhan 430083, China; 6 Department of Thyroid & Breast Surgery, Zigong First People’s Hospital, Zigong Academy of Medical Sciences, Zigong 643000, China; 7 Department of Integrated Chinese and Western Medicine, Zigong First People’s Hospital, Zigong Academy of Medical Sciences, Zigong 643000, China; 8 Department of Cardiology, Shanghai East Hospital, School of Medicine, Tongji University, Shanghai 200120, China; 9 Department of Nuclear Medicine, Sichuan Provincial People’s Hospital, University of Electronic Science and Technology of China, Chengdu 610072, China

**Keywords:** drug delivery system, biological sciences, cancer, biomaterials

## Abstract

Triple-negative breast cancer (TNBC) represents a highly aggressive subtype with limited treatment options, primarily relying on chemotherapy. Emerging therapies, including photothermal therapy (PTT) and related combination therapies, are considered highly promising. Naringenin, a flavonoid glycoside with diverse biological activities, can enhance immune responses as well as inhibit cancer cell proliferation. We developed photothermal bovine serum albumin (BSA)-biomineralized MnO_2_ nanoparticles (MnO_2_@BSA) by directly reducing KMnO_4_ with BSA, which were then combined with naringenin to achieve immuno-enhanced PTT for TNBC. The results show that MnO_2_@BSA exhibits strong light absorption and tumor-targeting capabilities. Naringenin and PTT together significantly suppress TNBC cell proliferation and migration while enhancing antitumor immune responses. *In vivo* experiments confirm that the combined therapy reduces tumor growth and metastatic risk compared to monotherapy. Additionally, the treatment exhibits a favorable safety profile, offering potential for improved clinical outcomes in breast cancer treatment.

## Highlights

• Naringenin acts as an immunoadjuvant to activate immune responses
• Photothermal therapy promotes immune cell infiltration into tumors
• The combined therapy reduces tumor growth and metastasis with a safety profile

## Graphical abstract

**Figure:**
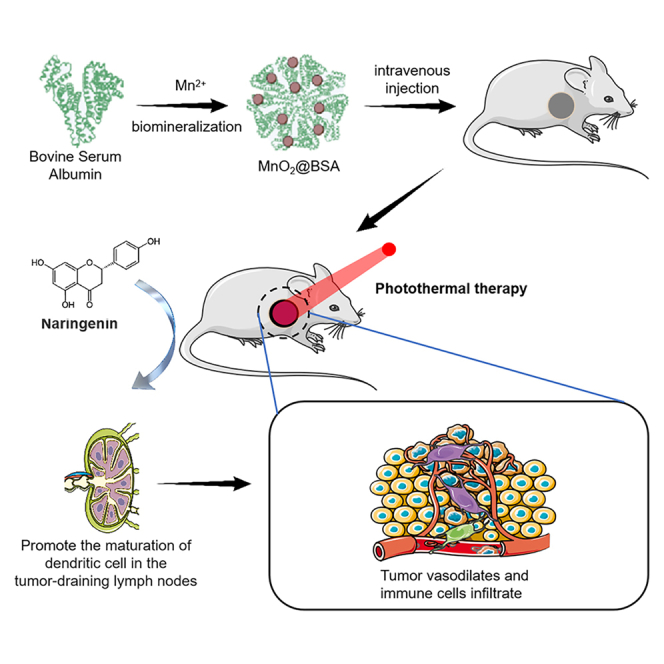

## Introduction

Triple-negative breast cancer (TNBC) is a highly invasive subtype of breast cancer, characterized by the absence of estrogen receptor, progesterone receptor, and human epidermal growth factor receptor 2. Treatment options for TNBC patients are limited, mainly relying on chemotherapy, resulting in significantly lower survival rates compared to patients with other types of breast cancer. Therefore, the development of innovative treatment strategies for TNBC is crucial.^1^^,^^2^

Photothermal therapy (PTT), a cancer treatment method that converts light energy into heat energy to directly kill tumor cells, minimizes damage to surrounding normal tissues.^2^^,^^3^ Its precision, non-invasiveness, and high controllability make it a promising strategy in cancer treatment. Studies have shown that PTT can disrupt the structure of tumors, facilitating the infiltration and activation of immune cells, thereby enhancing the ability of the immune system to clear tumors.^4^^,^^5^ In this study, photothermal bovine serum albumin (BSA)-biomineralized manganese dioxide nanoparticles (MnO_2_@BSA) were developed, showing excellent light absorption capabilities.

Albumin is a water-soluble protein widely present in mammalian plasma. It can bind with various substances, such as fatty acids, hormones, and drugs, preventing their premature degradation and transporting these target molecules through the bloodstream to different parts of the body, playing the role of a “natural drug delivery carrier” in the body.^6^^,^^7^ Studies have also shown that by exploiting the tumor’s preference for albumin uptake and the enhanced permeability and retention (EPR) effect, albumin bio-mineralized nanoparticles can be preferentially delivered to the tumor site and thus serve as an excellent photothermal therapeutic medium.^8^^,^^9^

Naringenin (NRG), a flavonoid glycoside primarily extracted from citrus fruits, has garnered extensive attention from the scientific community for its potent antioxidant, anti-inflammatory, and antitumor properties.^10^^,^^11^ It inhibits cancer cell proliferation through various mechanisms, including inducing apoptosis, interfering with the cell-cycle process, and inhibiting tumor cell invasion and migration. Furthermore, recent studies have revealed that NRG can activate the immune system, enhancing the body’s recognition and elimination of tumor cells.^12^

Although NRG has a certain therapeutic effect on tumors, it was found in our study that its therapeutic effect on TNBC was relatively poor at the treatment dose, failing to meet clinical requirements. Combining NRG with PTT to treat TNBC presents an innovative and potentially effective treatment strategy. The core idea is to utilize the immune-activating capabilities of NRG and the direct tumor-killing effect of PTT to achieve an effective treatment for TNBC.^13^^,^^14^ In this study, we verified that NRG combined with PTT has a good synergistic treatment effect on TNBC and further explored the possible reasons for the synergistic treatment of TNBC by PTT and NRG.

This combination therapy is expected to enhance the sensitivity of tumor cells to treatment and elicit a stronger immune response, effectively inhibiting the growth and spread of TNBC. Although the applications of NRG and PTT in cancer treatment have been extensively studied, their synergistic effects and mechanisms in treating TNBC have yet to be fully explored. This study aims to fill this knowledge gap by experimentally investigating the effects and potential mechanisms of the combined treatment of NRG and PTT for TNBC. Our research could not only provide a treatment option for TNBC patients but also pave the way for the integration of multimodal strategies in cancer therapy.

## Results

### Characterization of MnO_2_@BSA

**Figure 1:**
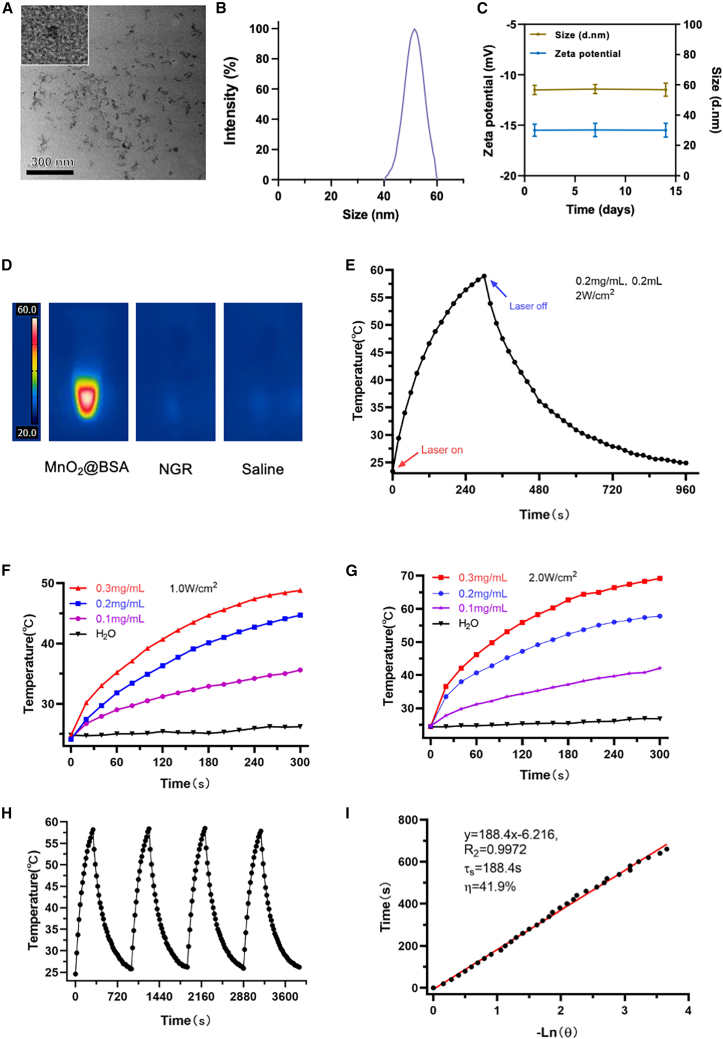
Characterization of MnO_2_@BSA nanoparticles (A) TEM image of MnO_2_@BSA nanoparticles (scale bars, 300 nm), with the inset showing a 5× magnified view. (B) Size distribution of MnO_2_@BSA nanoparticles measured by DLS. (C) Zeta potential and particle size of MnO_2_@BSA over a 15-day period (*n* = 3, data are represented as mean ± SD). (D) Temperature profile of MnO_2_@BSA under NIR laser irradiation. (E) Temperature response curves at different MnO_2_@BSA concentrations under 1 W/cm^2^ laser irradiation. (F) Temperature response curves at different MnO_2_@BSA concentrations under 2 W/cm^2^ laser irradiation. (G) Thermal imaging of MnO_2_@BSA, NGR, and saline solutions post-NIR irradiation. (H) Temperature changes of MnO_2_@BSA during multiple NIR laser on/off cycles. (I) Linear fitting for photothermal conversion efficiency (η) calculation.

In our study, MnO_2_@BSA nanoparticles were successfully synthesized, demonstrating a uniform distribution and well-defined morphology, as observed through transmission electron microscopy (TEM) (Figure 1A). Dynamic light scattering (DLS) analysis showed that the average particle size of MnO_2_@BSA was approximately 50 nm (Figure 1B), with minimal variation over a 15-day period, indicating stable particle size. Zeta potential measurements further confirmed the stability of MnO_2_@BSA, maintaining a stable surface charge throughout the same duration (Figure 1C).

Upon exposure to an 808 nm NIR laser (2 W/cm^2^), the temperature of the MnO_2_@BSA solution (0.2 mg/mL) continuously increased, reaching over 58°C within 300 s, and after the laser was turned off, the temperature gradually decreased back to baseline (Figure 1E). Additionally, the photothermal effect of MnO_2_@BSA exhibited clear concentration- and power-dependent characteristics: under irradiation at 1.0 W/cm^2^ (Figure 1F) and 2.0 W/cm^2^ (Figure 1G), MnO_2_@BSA solutions of varying concentrations (0.1, 0.2, and 0.3 mg/mL) showed significant temperature elevation, with higher concentrations and laser power resulting in greater temperature increases. In contrast, the control group (water) demonstrated minimal temperature changes under the same conditions, further confirming the effectiveness of MnO_2_@BSA as a photothermal agent.

The concentration-dependent photothermal effect was further explored at different laser power densities. At a concentration of 0.3 mg/mL, MnO_2_@BSA showed a robust temperature increase to nearly 70°C at 2 W/cm^2^ laser power, highlighting the strong capability of the nanoparticles to absorb and convert NIR light to heat (Figures 1F and 1G). MnO_2_@BSA demonstrated excellent photothermal stability, as evidenced by minimal changes in temperature during multiple on/off laser cycles (Figure 1H).

Additionally, the photothermal conversion efficiency of MnO_2_@BSA was calculated to be approximately 41.9%, confirming the effective energy transfer from NIR light to thermal energy (Figure 1I). These results suggest that MnO_2_@BSA has considerable potential for application in PTT.

### Cytotoxicity and photothermal efficacy on 4T1 cells

**Figure 2:**
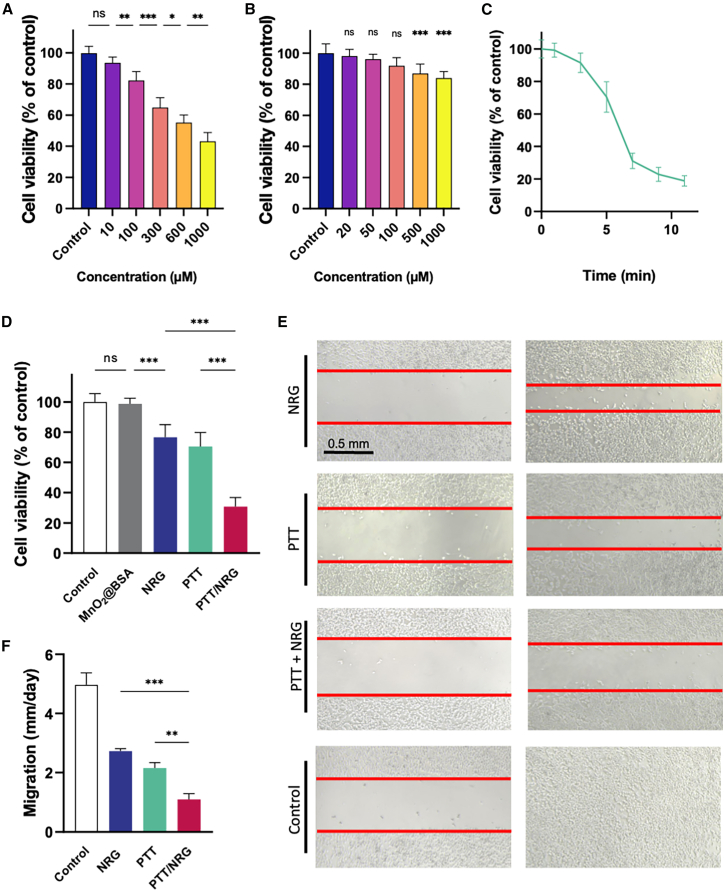
Cytotoxic effects of varying concentrations of NRG and MnO_2_@BSA on 4T1 TNBC cell viability (A) Cell viability of 4T1 cells after treatment with varying concentrations of NRG. (B) Cell viability of 4T1 cells treated with MnO_2_@BSA nanoparticles at different concentrations. (C) Cell viability of 4T1 cells treated with MnO_2_@BSA under NIR irradiation (100 μg/mL MnO_2_@BSA, 808 nm, 2 W/cm^2^, 5 min). (D) Cell viability of 4T1 cells after treatment with different groups: control, MnO_2_@BSA, NRG, PTT (MnO_2_@BSA + NIR). (E) Representative images of a wound healing assay showing the effects of different treatments on cell migration; scale bars, 0.5 mm. (F) Quantitative data of cell migration from the wound healing assay. ∗*p* < 0.05, ∗∗*p* < 0.01, ∗∗∗*p* < 0.001, ns *p* ≥ 0.05. Data are represented as mean ± SD, and all experiments were conducted with *n* = 6 per group.

We evaluated the cytotoxic effects of the drug NRG on 4T1 TNBC cells at varying concentrations. The cell viability assay demonstrated a dose-dependent decrease in cell viability as the concentration of NRG increased from 10 to 1,000 μM (Figure 2A). In a similar assay, MnO_2_@BSA nanoparticles were tested at concentrations ranging from 20 to 1,000 μg/mL. The results indicated that MnO_2_@BSA nanoparticles did not exhibit significant cytotoxicity at concentrations up to 100 μg/mL, suggesting no toxicity within this concentration range (Figure 2B).

The photothermal efficacy of MnO_2_@BSA nanoparticles (100 μg/mL) was significantly enhanced when combined with NIR light. During a 5-min NIR treatment period, a pronounced decrease in cell viability was observed, highlighting the potent photothermal effect of the nanoparticles on 4T1 TNBC cells (Figure 2C). This demonstrates that MnO_2_@BSA nanoparticles are inherently biocompatible without NIR light but become highly effective in killing cancer cells upon photothermal activation.

Comparative assessments of cell viability across different treatment groups—control, MnO_2_@BSA, NRG, PTT, and the combinatory PTT/NRG—revealed distinct outcomes. The control and MnO_2_@BSA groups exhibited high cell survival rates, indicating minimal inherent cytotoxicity. NRG alone showed moderate toxicity, while the PTT group demonstrated a significant reduction in cell viability. The combination of PTT with NRG further amplified this effect, resulting in a marked decrease in cell viability (Figure 2D). This suggests a synergistic effect of NRG in enhancing the efficacy of PTT for tumor therapy.

The migration capacity of 4T1 TNBC cells subjected to different treatments was also evaluated. The combination of PTT with NRG significantly reduced cell migration compared to the control, NRG alone, and PTT alone groups. These results indicate that the combined treatment not only decreases cell viability but also significantly inhibits the migratory ability of cancer cells, potentially impeding metastatic progression (Figures 2E and 2F).

### Efficacy of NRG and MnO_2_@BSA-enhanced photothermal therapy in a 4T1 tumor model

**Figure 3:**
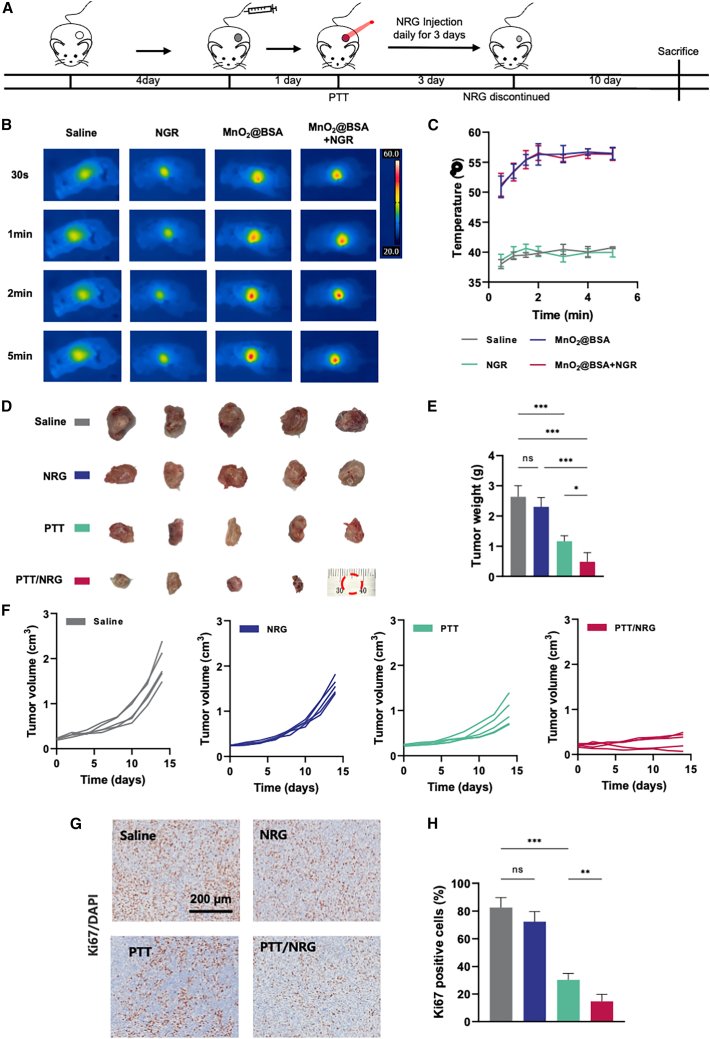
Efficacy of NRG- and MnO_2_@BSA-enhanced photothermal therapy in a 4T1 tumor model (A) Schematic representation of the treatment timeline for the 4T1-tumor-bearing mouse model. (B) Thermal imaging of tumor sites in different treatment groups (saline, NRG, MnO_2_@BSA, and MnO_2_@BSA+NRG) at various time points post-laser irradiation (30 s, 1 min, 2 min, and 5 min). (C) Temperature change curves for tumor sites during laser irradiation in different groups. (D) Photographs of excised tumors from different treatment groups. (E) Tumor weight measurements of excised tumors from each group. (F) Tumor volume growth curves for saline, NRG, PTT, and PTT/NRG groups over the observation period. (G) Ki67/DAPI staining of tumor sections to assess cell proliferation in each group; scale bars, 200 μm. (H) Quantification of Ki67-positive cells in tumor sections from different treatment groups. ∗*p* < 0.05, ∗∗*p* < 0.01, ∗∗∗*p* < 0.001, ns *p* ≥ 0.05. Data are represented as mean ± SD, and all experiments were conducted with *n* = 5 animals per group.

In the 4T1-tumor-bearing mouse model, PTT was initiated 24 h after the administration of MnO_2_@BSA nanoparticles. Following laser irradiation, intraperitoneal NRG injections were administered daily for three consecutive days to form the PTT/NRG treatment group. In comparison, the PTT group received no additional NRG treatment, and the NRG group was not subjected to laser irradiation. Tumor size and weight were carefully monitored post-treatment to assess the efficacy of each therapeutic approach (Figure 3A).

Thermal imaging demonstrated that MnO_2_@BSA treatment significantly elevated tumor temperature under laser irradiation. Specifically, both the MnO_2_@BSA group and the MnO_2_@BSA+NRG group exhibited markedly higher tumor temperatures compared to the saline and NRG groups, indicating the effective photothermal conversion capability of MnO_2_@BSA. Post-treatment, tumors were excised and compared visually and by weight. The PTT/NRG combination therapy resulted in the smallest tumor sizes and the lowest tumor weights, indicating a significant reduction in tumor growth compared to the saline control, NRG monotherapy, and PTT alone (Figures 3D and 3E).

Tumor volume measurements over time showed that saline-treated mice had progressive and unchecked tumor growth. In contrast, NRG and PTT monotherapies demonstrated moderate tumor growth inhibition. The combination of PTT with NRG significantly suppressed tumor growth, resulting in the smallest tumor volumes throughout the observation period (Figure 3F). These results highlight the enhanced therapeutic effect of combining NRG with PTT compared to either treatment alone.

Ki67 staining, a marker for cell proliferation, was conducted on tumor sections. The analysis revealed high proliferation rates in the saline-treated group, moderate rates in the NRG and PTT monotherapy groups, and significantly reduced proliferation in the PTT/NRG combination group, as indicated by the percentage of Ki67-positive cells (Figures 3G and 3H). This suggests that the combination treatment not only inhibits tumor growth but also effectively suppresses tumor cell proliferation.

### Network pharmacological analysis of NRG in the treatment of TNBC

**Figure 4:**
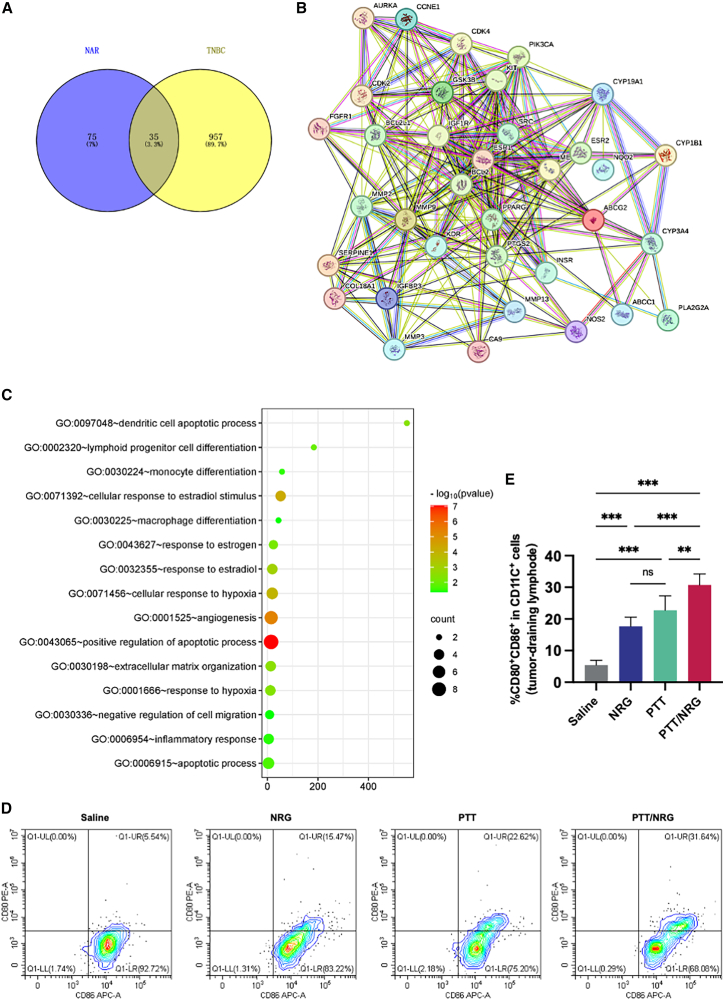
Network pharmacological analysis (A) Venn diagram showing the numbers of the overlapping genes between NRG and TNBC. (B) The PPI network of the overlapping targets. (C) GO enrichment analysis of the biological process overlapping targets. (D) Representative flow cytometry plots of CD80^+^CD86^+^ expression in CD11c^+^ cells from tumor-draining lymph nodes of 4T1-tumor-bearing mice treated with different regimens. (E) Quantitative analysis of CD80^+^CD86^+^ expression in CD11c^+^ cells. Data are presented as mean ± SD. ∗*p* < 0.05, ∗∗*p* < 0.01, ∗∗∗*p* < 0.001, ns *p* ≥ 0.05. Data are represented as mean ± SD, and all experiments were conducted with *n* = 5 animals per group.

Network pharmacology is a bioinformatics method to analyze the basic mechanism of action of drugs on diseases.^15^^,^^16^^,^^17^^,^^18^ As shown in Figure 4A, by defining the interaction between the 992 target genes associated with TNBC and the 110 target genes associated with NRG, we got 35 different genes, which were considered as the key targets in the treatment of TNBC. We then input these genes into the STRING database to build a protein-protein interaction (PPI) network (Figure 4B). In order to reveal the biological characteristics of 162 intersecting target genes, we performed gene ontology (GO) enrichment analysis using the Metascape tool and selected enrichment results under the conditions of *p* < 0.01, minimum enrichment >1.5, and minimum overlap of 3. Figure 4C shows the top 15 terms that are significantly enriched in terms of biological processes. The GO enrichment analysis shows significant enrichment of immune-related pathways. Notably, dendritic cell apoptotic process (GO:0097048) and lymphoid progenitor cell differentiation (GO:0002320) strongly suggest that NRG may enhance the activation of tumor-draining lymph nodes (TDLNs) and promote dendritic-cell-mediated antigen presentation, which are critical steps for initiating tumor-specific adaptive immune responses.^19^^,^^20^ Enrichment in monocyte differentiation (GO:0030224), macrophage differentiation (GO:0030225), and inflammatory response (GO:0006954) further supports the possibility that NRG modulates the tumor microenvironment toward an immune-active state. Therefore, we conclude that an important therapeutic effect of NRG on TNBC comes from the activation of tumor-specific immune function, and it is consistent with many reported findings that NRG can act as an immunoadjuvant.^21^^,^^22^

To validate the maturation of TDLNs triggered by NRG, tumor-draining lymph nodes were collected and analyzed by flow cytometry on the 6th day after different treatments. The results showed that NRG did act as an immunoadjuvant and successfully up-regulated the number of mature dendritic cells (Figures 4D and 4E). In the results, we also found that PTT also had the function of up-regulating the number of mature dendritic cells (Figures 4D and 4E). According to the reports, we believed that this was caused by PTT leading to tumor death and the leakage of tumor immune-related contents.^13^^,^^23^^,^^24^

### Immune response evaluation in 4T1-tumor-bearing mice treated with PTT and NRG

**Figure 5:**
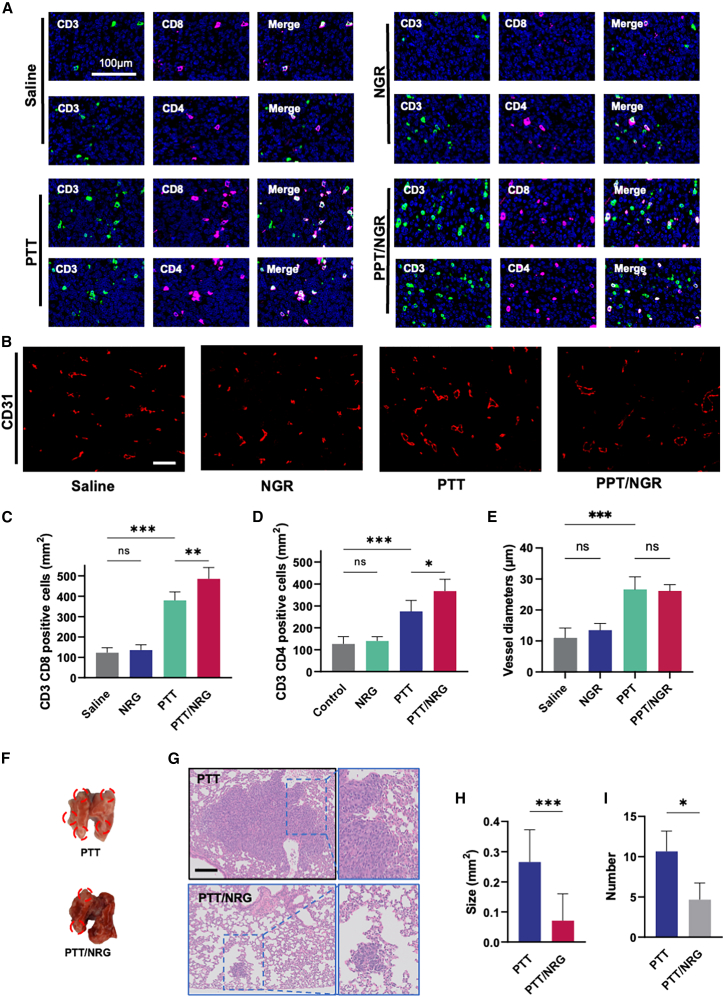
Analysis of immune cell infiltration, tumor vasculature, and metastasis in 4T1 tumor model (A) Immunofluorescence staining of tumor sections for CD3, CD8, and CD4 T cell markers in different treatment groups; scale bars, 100 μm. (B) CD31 immunofluorescence staining to visualize blood vessels in tumor tissues from different treatment groups. (C) Quantification of CD8^+^ T cells in tumor sections for each group; scale bars, 100 μm. (D) Quantification of CD4^+^ T helper cells in tumor sections for each group. (E) Quantification of blood vessels in tumor tissues from different treatment groups. (F) Direct observation of lung tissue excised from tumor-bearing mice that received additional tail vein injections of 4T1 cells after initial photothermal treatment, with red circles highlighting the visibly identifiable grayish metastatic tumor tissue. (G) Representative images of lung tissue sections stained with H&E to evaluate metastatic burden; scale bars, 100 μm. (H) Quantification of the average size of metastatic lesions in the lungs. (I) Quantification of the number of metastatic nodules in the lungs from PTT and PTT/NRG groups. ∗*p* < 0.05, ∗∗*p* < 0.01, ∗∗∗*p* < 0.001, ns *p* ≥ 0.05. Data are represented as mean ± SD, and all experiments were conducted with *n* = 5 animals per group.

We conducted a further evaluation of the tumor tissue using immunofluorescence staining (Figures 5A and 5B). Immunofluorescence staining for T cell markers CD3 and CD8, as well as T helper cell markers CD3 and CD4, was performed on tumor sections from saline-, NRG-, PTT-, and PTT/NRG-treated groups.^25^ The merged images show that the combined PTT/NRG treatment significantly increased the infiltration of both CD8^+^ cytotoxic T cells and CD4^+^ T helper cells compared to the other treatments (Figure 5A). Quantitative analysis demonstrated that the PTT/NRG group had a higher infiltration of CD8^+^ T cells within the tumor microenvironment than the saline and NRG groups and a significant increase compared to PTT alone (Figure 5C). Similarly, the combined PTT+NRG treatment led to an enhanced presence of CD4^+^ T helper cells within the tumors compared to the control group, indicating an improved immune response (Figure 5D).

NRG, in combination with PTT, showed improved infiltration of immune cells. However, there was no significant difference between the NRG group and the saline group. It has been reported that ICG-mediated PTT can destroy the original vasculature of tumors and dilate tumor blood vessels, thereby enhancing the therapeutic effect of CAR-T on solid tumors.^5^ We speculated that MnO_2_@BSA-mediated PTT may alter the tumor microenvironment in this way, thereby enhancing the efficacy of NRG in tumor treatment. For this reason, tumor vascular immunofluorescence imaging using CD31 as a marker was performed on the tumor (Figure 5B).^26^ We found that blood vessels in the collapsed tumor were significantly dilated in the PTT group and PTT/NRG group (Figure 5E). The result helps explain why NRG alone activated the body’s anti-tumor immunity but did not increase the number of infiltrating immune cells in the tumor. It also helps explain why NRG alone is relatively ineffective in treating TNBC.

Figure 5F shows a direct observation of lung tissue excised from tumor-bearing mice that received additional tail vein injections of 4T1 cells after initial photothermal treatment. Compared to the PTT group, the PTT/NRG group exhibited significantly reduced metastatic foci, indicating a lighter metastatic burden. Hematoxylin and eosin (H&E) staining of lung tissue sections further confirmed that the PTT/NRG group showed fewer metastatic histopathological features compared to the PTT group, indicating a reduction in metastatic burden (Figure 5G). The average size of lung metastatic foci in the PTT+NRG group was significantly smaller than that in the PTT group alone, suggesting that the combined treatment effectively restricted the progression of metastatic tumor growth (Figure 5H). Moreover, the number of lung metastatic nodules in the PTT+NRG treatment group was lower than in the PTT group (Figure 5I). These findings suggest that the immune adjuvant effect of NRG, combined with the tumor antigen release effect of PTT, can enhance the body’s antitumor immunity, thereby reducing the frequency of metastatic tumor formation.^21^^,^^22^^,^^27^

### Safety profile of PTT and NRG treatments in 4T1-tumor-bearing mice

**Figure 6:**
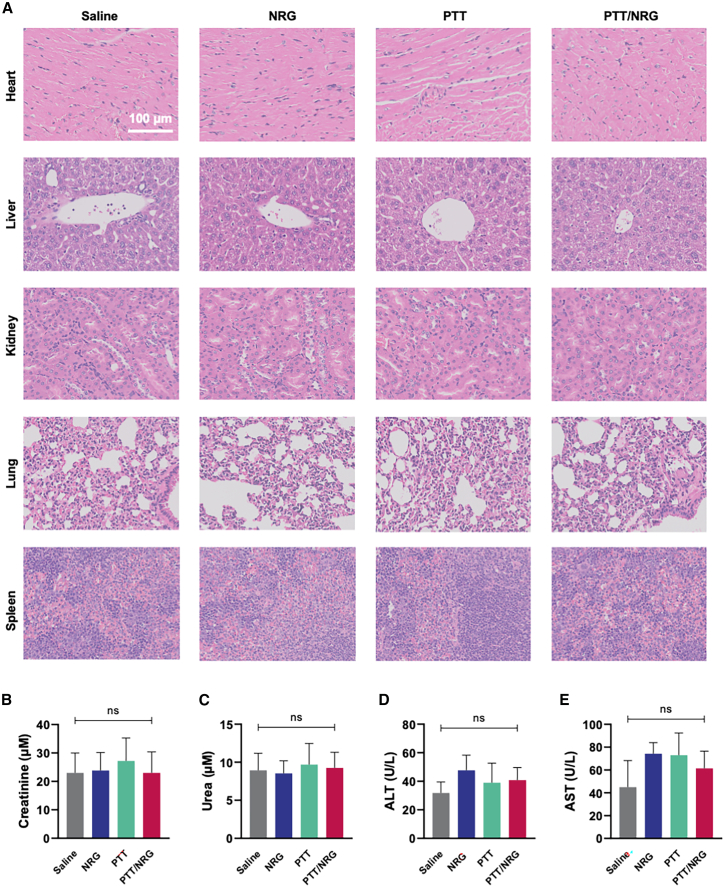
Biosafety of treatment (A) Histopathological analysis of heart, liver, kidney, lung, and spleen tissues across saline-, NRG-, PTT-, and PTT/NRG-treated groups, stained with hematoxylin and eosin (H&E) to evaluate systemic toxicity; scale bars, 100 μm. (B) Serum creatinine levels for renal function assessment. (C) Serum urea concentrations as an additional renal function parameter. (D) Alanine aminotransferase (ALT) levels, an indicator of liver function. (E) Aspartate aminotransferase (AST) measurements to complement the liver function profile. ∗*p* < 0.05, ∗∗*p* < 0.01, ∗∗∗*p* < 0.001, ns *p* ≥ 0.05. Data are represented as mean ± SD, and all experiments were conducted with *n* = 5 animals per group.

Histological analysis via H&E staining of critical organs, including the heart, liver, kidney, lung, and spleen, revealed no substantial differences among the saline, NRG, PTT, and PTT/NRG groups, suggesting that the treatments did not induce discernible organ damage, and the organ structures remained intact without evidence of tissue injury (Figure 6A). Consistent with the histopathological findings, the evaluation of kidney and liver function through serum biomarkers, specifically creatinine, urea, ALT, and AST levels, showed no significant alterations across all treatment groups (Figures 6B–6E). These biochemical assays further substantiate the histological evidence, indicating a favorable safety profile for both PTT and NRG treatments when applied individually or in combination.

## Discussion

Our research provides substantive evidence that combining PTT with the chemotherapeutic agent NRG markedly enhances therapeutic efficacy against TNBC in a mouse model. The combinatorial approach not only impedes tumor growth and metastasis but also promotes a favorable immune response without inducing significant systemic toxicity.

Recent advances have increasingly highlighted the importance of TME remodeling in improving immunotherapy outcomes, especially regarding the spatial distribution and infiltration capacity of immune cells. In our study, the PTT-induced remodeling of tumor architecture appears to play a pivotal role in overcoming these immune-exclusion barriers. Furthermore, the spatial pattern of gene expression within the TME—previously considered a limiting factor in effective immunomodulation—may be favorably altered by this combination strategy. These mechanisms likely contribute to the observed synergy between PTT and chemotherapy.^28^^,^^29^ While NRG alone showed limited benefits, likely due to the structural complexity of solid tumors impeding drug delivery and immune cell infiltration, the addition of PTT disrupted these barriers.^28^^,^^29^ This disruption facilitated NRG delivery and action at the tumor site and may have contributed to the release of tumor antigens, thus enhancing the immunogenicity of the tumor. As a result, the combination therapy led to a significant recruitment of immune cells, particularly cytotoxic T cells, to the tumor microenvironment. Our study underscores the potential of integrating PTT with conventional chemotherapy to surmount the challenges posed by the tumor microenvironment. By leveraging the dual mechanisms of direct tumor ablation and enhanced immune response, this strategy shows promise for improving outcomes in patients with TNBC, a variant of breast cancer notorious for its aggressive nature and limited treatment options. Future research should aim to further elucidate the mechanisms behind the observed synergy, optimize treatment parameters, and evaluate the long-term efficacy and safety of this therapeutic strategy. The insights gained from this study have the potential to inform the development of more effective, immune-responsive cancer therapies, offering hope for improved patient prognoses.

### Limitations of the study

Several limitations should be acknowledged in this study. First, although the combination of NRG and photothermal therapy demonstrated significant antitumor and immunomodulatory effects *in vitro* and *in vivo*, the experiments were primarily conducted in murine 4T1 tumor models, which may not fully recapitulate the heterogeneity and complexity of human triple-negative breast cancer. Second, the pharmacokinetics, biodistribution, and metabolic stability of NRG and MnO_2_@BSA nanoparticles were not systematically evaluated, and these factors could influence therapeutic efficacy and safety in clinical applications. Third, while the study highlighted immune activation, the specific immune pathways and cell subsets responsible for the observed effects require further mechanistic validation through in-depth immunological assays and genetic models. Finally, long-term safety, potential immunotoxicity, and the impact of repeated administration remain to be clarified before translation to clinical settings. Addressing these limitations will be crucial for advancing this combination strategy toward clinical application.

## Resource availability

### Lead contact

Further information and requests for resources and reagents should be directed to and will be fulfilled by the lead contact, Fuqiang Shao (m15228213350@163.com).

### Materials availability

All unique reagents generated in this study are available from the lead contact.

### Data and code availability

• Any additional information required to reanalyze the data reported in this article is available from the lead contact upon request.
• This article does not report original code.
• The experimental protocols and other materials in this study are available from the lead contact.

## Acknowledgments

This work was supported by the 10.13039/501100018542Natural Science Foundation of Sichuan (2024NSFSC1787), Scientific Research Project of Zigong Health Commission (22yb051), Youth Innovation Project of Sichuan Medical Association (Q22013), and 10.13039/501100001809National Natural Science Foundation of China (no. 823 02238).

## Author contributions

C.L., Y.Y., and Q.X. contributed equally to this work. C.L. and Y.Y. conceived and designed the project. Y.Y., Q.X., C.Z., N.L., H.L., S.Y., H.W., and X.P. carried out the experiments. C.L., Y.Y., and Q.X. analyzed the data. Y.Y. and C.Z. drafted the manuscript. Z.Z. and F.S. supervised the research and revised the manuscript. F.S. and K.Z. provided the funding. All authors read and approved the final manuscript.

## Declaration of interests

The authors declare no competing interests.

## STAR★Methods

### Key resources table

**Table:** 

REAGENT or RESOURCE	SOURCE	IDENTIFIER
**Antibodies**		

Ki-67 Polyclonal antibody	Proteintech	28074-1-AP; RRID: AB_3695704
CD80 Polyclonal antibody	Proteintech	14292-1-AP; RRID: AB_10640809
CD86 Recombinant monoclonal antibody	Proteintech	83213-5-RR; RRID: AB_3670898
CD3 Polyclonal antibody	Proteintech	17617-1-AP; RRID: AB_10492732
CD4 Polyclonal antibody	Proteintech	19068-1-AP; RRID: AB_10603357
CD31 Polyclonal antibody	Proteintech	28083-1-AP; RRID: AB_2881055

**Chemicals, peptides, and recombinant proteins**		

Naringenin	Yuanye	B21596; CAS: 480-41-1
bovine serum albumin	Solarbio	A8010; CAS: 9048-46-8
KMnO4	Aladdin	P112386; CAS: 7722-64-7

**Critical commercial assays**		

MTT Assay Kit	Solarbio	M1020

**Experimental models: Cell lines**		

Mouse: 4T1 cells	Procell	CL-0007

**Experimental models: Organisms/strains**		

Mouse: C57BL/6JNifdc	Charles River	C57BL/6JNifdc

**Software and algorithms**		

ImageJ	Schneider et al.	https://imagej.nih.gov/ij/
GraphPad Prism	GraphPad Software	https://www.graphpad.com/

### Experimental model and study participant details

#### Cell lines

4T1 TNBC cells were cultured in RPMI-1640 medium supplemented with 10% fetal bovine serum (FBS) and 1% penicillin-streptomycin. The cell lines were sourced from Procell (Wuhan, China). The supplier provided an authentication report for the cell lines, and they were tested for mycoplasma contamination, which was confirmed to be negative.

#### Animal models

Female C57BL/6 mice, 6 weeks old, 20g ± 1g, were purchased from Charles River (Chengdu, China). Tumor-bearing mouse models were created by subcutaneously injecting 1 × 10^∧^6 4T1 TNBC cells suspended in 100 μL PBS into the back of the mice. All mice were housed in standard animal facilities with a 12-hour light/dark cycle, a temperature of 24°C, and provided with standard diet. All animal experiments complied with the guidelines approved by the Animal Care and Use Committee of Sichuan Provincial People's Hospital (Approva249 No. 202S191).

### Method details

#### Synthesis of nanoparticles and characterization

The protocol for synthesizing MnO_2_@BSA nanoparticles involves a straightforward *in situ* redox reaction. This process starts by mixing a solution of BSA with potassium permanganate (KMnO_4_) under stirring. BSA acts as a reductant and stabilizing template. Once the reaction occurs, indicated by a color change signaling MnO_2_ nanoparticle formation, the mixture is centrifuged to separate the nanoparticles. The precipitate is then washed to remove any residual reactants and dried for subsequent use and analysis. MnO_2_@BSA were characterized using TEM (Hitachi, Japan) and DLS analysis (Malvern Instruments Ltd, Worcestershire, UK). To assess the stability of MnO_2_@BSA, the hydrodynamic diameters were monitored for 14 days using a DLS system. The photothermal properties of MnO_2_@BSA were evaluated using a NIR laser (808 nm) at different power densities. The temperature changes were monitored using an infrared thermal camera to assess the photothermal conversion efficiency. The concentration-dependent photothermal effects were also investigated by varying the concentration of MnO_2_@BSA nanoparticles and measuring the temperature increase under laser irradiation.

The photothermal conversion efficiency (η) of MnO_2_@BSA nanoparticles was calculated based on the temperature change data during laser irradiation. The calculation involved recording the maximum temperature achieved during irradiation and the cooling rate after the laser was turned off. The efficiency was determined using the formula: η = (γ ΔT_max - Q_dis)/I(1 - 10^∧^(-A808)), where γ is the thermal constant, ΔT_max is the temperature change, Q_dis is the heat dissipated by the solvent, I is the laser power density, and A808 is the absorbance of MnO_2_@BSA at 808 nm. The cooling curve was used to determine γ, and the efficiency was calculated as described in previously published.

#### Cell culture and treatment

Cells were seeded in 96-well plates at a density of 5,000 cells per well and allowed to adhere overnight. For cytotoxicity assays, cells were treated with different concentrations of NRG (100-400 μM) or MnO_2_BSA nanoparticles (20-100 μg/mL) for 24 hours.

#### Cell viability assay

Cell viability was assessed using the MTT assay. After treatment, MTT reagent (5 mg/mL) was added to each well, and the plates were incubated for 4 hours at 37°C. Formazan crystals formed by metabolically active cells were dissolved in dimethyl sulfoxide, and absorbance was measured at 570 nm using a microplate reader. Cell viability was calculated as a percentage of the control group.

#### Photothermal treatment *in vitro*

For photothermal experiments, MnO_2_@BSA-treated cells were exposed to an 808 nm NIR laser at different power densities for 5 minutes. The temperature of the cell culture medium was monitored using an infrared thermal camera. Cell viability post-irradiation was determined using the MTT assay as described above.

#### Wound healing assay

To evaluate the effect of treatments on cell migration, a wound healing assay was performed. 4T1 cells were seeded in 6-well plates and grown to 90% confluency. A scratch was made using a sterile pipette tip, and cells were treated with NRG, MnO_2_@BSA, PTT, or PTT/NRG. Images of the wound area were captured at 0 and 24 hours, and the wound closure was quantified using ImageJ software.

#### Migration assay

The migration of 4T1 cells was further assessed using a transwell migration assay. Cells were seeded in the upper chamber of transwell inserts with serum-free medium, and the lower chamber contained medium with 10% FBS as a chemoattractant. After 24 hours of incubation, cells that migrated to the lower surface were fixed, stained, and counted under a microscope.

#### Treatment to breast cancer mouse models

The NGR dosage for treating mice is administered intraperitoneally at 100 mg/kg based on body weight. This dosage was determined through a comprehensive review of the literature, considering both the safety dosage of NGR and the commonly used dosages for treating mice.^30^^,^^31^^,^^32^ 4T1 tumor-bearing mice were allocated randomly into 4 groups, each consisting of 5 mice. The groups received the following treatments: normal saline (saline group), MnO_2_@BSA at a dose of 1 mg kg^−1^ with laser (PTT group), MnO_2_@BSA at a dose of 1 mg kg^−1^ with laser and followed by NRG therapy at a dose of 1mg/kg per day for 3 days (PTT/NRG). MnO_2_@BSA at a dose of 1 mg kg^−1^ without laser treatment and followed by NRG therapy at a dose of 1mg/kg per day for 3 days (NRG). The PTT and PTT/NRG group receiving laser treatment were anesthetized using 2% isoflurane, and their tumor sites were subjected to 1.4 W·cm^−2^ 808 nm laser irradiation for 5 minutes. The changes in temperature were monitored using a thermal imaging camera. Subsequently, the tumor sizes and body weights of the mice were measured over 14 days. On the 14th day, all the mice were euthanized. The tumor tissues were collected, weighed, photographed, and preserved for further histological examinations. Immunohistochemistry was conducted to evaluate the Ki67 positive cells, CD3^+^CD4^+^ and CD3^+^CD8^+^ positive T cells.

#### Immunohistochemistry for the assessment of tumor vessel

The assessment of the tumor vessel after treatments involved collecting heart samples from the 4T1 tumor-bearing mouse. Tumors from various groups were initially fixed in a 4 wt% paraformaldehyde solution for 24 hours. Afterward, a process of dehydration through gradient ethanol concentrations and xylene-based transparency was carried out. The resultant samples were sectioned to a thickness of 4 μm on slides. This included steps such as rehydration, treatment with citrate buffer under high-temperature microwave conditions, permeabilization using 0.3% Triton X-100 for 30 minutes, and blocking with 1% (w/w) bovine serum for 30 minutes. The tumor sections were then exposed to primary antibodies targeting endothelial cell (rabbit anti-CD31) overnight at 4°C. Following this, the sections were washed with phosphate buffer saline and subsequently incubated with Cy3-labeled secondary antibodies (goat anti-rabbit IgG from BosterBio) for 90 minutes.

#### *In vivo* toxicity evaluation

After euthanizing the mouse, whole blood was collected, and serum creatinine, BUN, ALT, and AST levels were measured using a biochemical analyzer (Chemray240, China). Major organs (heart, liver, lung, kidney, spleen) were harvested for H&E staining (Hematoxylin and Eosin Staining Kit, Beyotime, Shanghai, China).

### Quantification and statistical analysis

Data are expressed as mean ± standard deviation (SD). Group comparisons were conducted using an unpaired Student's t-test. Statistical significance was defined as P < 0.05. P-values are categorized as follows: ∗P < 0.05, ∗∗P < 0.01, ∗∗∗P < 0.001, ns P ≥ 0.05. All statistical analyses were performed using GraphPad Prism version 8.0 software.
